# First insights into the microbial diversity in the omasum and reticulum of bovine using Illumina sequencing

**DOI:** 10.1007/s13353-014-0258-1

**Published:** 2015-01-21

**Authors:** Shuai Peng, Jigang Yin, Xiaolei Liu, Boyin Jia, Zhiguang Chang, Huijun Lu, Ning Jiang, Qijun Chen

**Affiliations:** 1 Key Laboratory of Zoonosis, Ministry of Education, College of Veterinary Medicine, Jilin University, Xi An Da Lu 5333, Changchun, 130062 China; 2 College of Food Science and Engineering, Jilin Agricultural University, Xin Cheng Da Jie 2888, Changchun, 130118 China

**Keywords:** Illumina, Microbiome diversity, Omasum, Rumen, Reticulum, V3 region of the 16S rRNA

## Abstract

The digestive systems of mammals harbor a complex gut microbiome, comprising bacteria and other microorganisms that confer metabolic and immunological benefits to the host. Ruminants that digest plant-based foods have a four-compartment stomach consisting of the rumen, reticulum, omasum, and abomasum. The microorganisms in the stomach are essential for providing the host with critical nutrients. However, the majority of these microorganisms are unknown species. The microbiome of the stomach is diverse, and the majority of these organisms cannot be cultured. Next-generation sequencing (NGS) combined with bioinformatic analysis tools have allowed the dissection of the composition of the microbiome in samples collected from a specific environment. In this study, for the first time, the bacterial composition in two compartments, the reticulum and the omasum, of bovine were analyzed using a metagenomic approach and compared to the bacterial composition of the rumen. These data will assist in understanding the biology of ruminants and benefit the agricultural industry. The diversity and composition of the bacterial community in samples collected from the rumen, reticulum, and omasum of bovines in the Changchun Region of Northeast China were analyzed by sequencing the V3 region of the 16S rRNA gene using a barcoded Illumina paired-end sequencing technique, and the primary composition of the microbiome in the rumen, reticulum, and omasum of the bovines was determined. These microbiomes contained 17 phyla and 107 genera in all three samples. Five phyla, Bacteroidetes, Firmicutes, Proteobacteria, Spirochaetes, and Lentisphaerae, were the most abundant taxonomic groups. Additionally, the different stomach compartments harbored different compositions of the microorganisms.

**Electronic supplementary material:**

The online version of this article (doi:10.1007/s13353-014-0258-1) contains supplementary material, which is available to authorized users.

## Introduction

The rumen is a complex anaerobic microbial ecosystem in ruminants, and it is able to digest a range of plant materials by virtue of its large population of microbes, which include bacteria, archaea, fungi, and protozoa. These microbes influence the health of the ruminant and provide the host with nutrients that are predominantly in the form of volatile fatty acids and microbial proteins (Hess et al. 2011; Li et al. 2011). Additionally, the efficient microbial transformation of plant fibers results in the production of meat and milk for human consumption.

The rumen and the three other stomach compartments, the reticulum, the omasum, and the abomasum, are important colonization sites for many commensal microorganisms. Numerous studies have been performed to obtain a better understanding of the composition of rumen microbial communities (Jami et al. 2014; Li et al. 2012a; Zhou and Guan 2014), whereas limited effort has been undertaken to characterize the microbiota in the three other stomachs. Culture-independent methods, such as the sequencing of 16S rDNA gene libraries, single-strand conformation polymorphism (SSCP), random amplified polymorphic DNA (RAPD), and denaturing gradient gel electrophoresis (DGGE) separation of polymerase chain reaction (PCR)-amplified 16S rDNA fragments, have generated a tremendous amount of additional information regarding the microbial communities in the rumen (Cappa et al. 2014; Miteva et al. 2014). Metagenomics studies using next-generation sequencing (NGS) technologies provide us with an unprecedented opportunity to conduct in-depth sequencing and data analysis on samples derived from any environment, including the rumen microbiota, at a deeper level than has been previously performed (Breakwell et al. 2014; Be et al. 2014; Solomon et al. 2014). The hypervariable V3 region of the 16S ribosomal RNA gene contains the maximum nucleotide heterogeneity that has been selected for the analysis of microbiota in several studies (Huse et al. 2008; Li et al. 2014; Wang et al. 2014). The goal of the current study was to describe the characteristics of the composition and the structure of the microbial communities in the bovine stomachs.

## Materials and methods

### Sample collection and DNA extraction

The samples were collected from a slaughterhouse in Changchun, China. After the bovines were butchered, the contents in the stomachs (the rumen, reticulum, omasum, and abomasum) were immediately collected from three adult Simmentals fed with dried distillers’ grains containing soluble and corn silage. Briefly, the samples were suspended in physiological saline, vortexed vigorously for 3 min, and passed through four layers of medical gauze to remove any food particles, plant remnants, and other undigested materials. The rumen, reticulum, and omasum samples from the different animals were each combined. The bacteria were pelleted by centrifugation and resuspended in a saline solution. The DNA from the bacteria in the rumen, reticulum, omasum, and abomasum was extracted separately using the QIAamp DNA Stool Kit (Qiagen, Hilden, Germany). Our study was reviewed and approved by the Ethics Committee of Jilin University (ethical clearance application number IZ-2009-III). All of the animal work was conducted according to Chinese and international guidelines.

### PCR amplification and Solexa GAII sequencing

PCR primers (338F and 533R) were designed to amplify the conserved V3 regions of the 16S rDNA. The sequence of 338F is 5′-ACTCCTACGGGAGGCAGCAG-3′ and the sequence of 533R is 5′-TTACCGCGGCTGCTGGCAC-3′ (Huse et al. 2008). Each 25-μl PCR reaction mixture contained 2.5 μl of TaKaRa 10× Ex Taq buffer (Mg^2+^ free), 2 μl of deoxynucleoside triphosphate (dNTP) mix (2.5 mM each), 1.5 μl of Mg^2+^ (25 mM each), 0.25 μl of TaKaRa Ex Taq DNA polymerase (2.5 U), 1 μl of template DNA, 0.5 μl of 10 μM barcode primer 338F, 0.5 μl of 10 μM primer 533R, and 16.75 μl of double-distilled H_2_O. The thermal cycling consisted of 3 min at 94 °C and 25 cycles consisting of 30 s at 94 °C, 45 s at 57 °C, and 1 min at 72 °C, followed by 7 min at 72 °C. The amplified products were purified using the QIAquick PCR Purification Kit (Qiagen). The DNA was end repaired, poly-A tailed, and paired-end (PE) adaptor ligated using the Paired-End Library Preparation Kit (Illumina). After ligation of the adaptors, the sample was purified and dissolved in 30 μl of elution buffer, and 1 μl of the mixture was used as a template for 12 cycles of PCR amplification using primers matched to the adaptor sequences (Zhou et al. 2011). The PCR products were gel purified using the QIAquick Gel Extraction Kit (Qiagen) and sequenced using the 150-bp PE strategy on an Illumina GAII system according to the manufacturer’s instructions.

### Data analysis

The barcode Illumina PE sequencing (BIPES) method was used to process the raw sequences (Zhou et al. 2011). Then, the PE reads were assembled based on the overlapping sequence regions using the OVERLAP software program, with the criteria that at least 30 bp overlapped and no mismatches were permitted in the region. To collect more sequence tags, we trimmed 3 bp from the end of the reads that did not satisfy the above rule and repeated the overlapping process until no reads could be overlapped. After the overlapping, the sequences that were less than 55 bp were removed. The 3′ end of the primer sequences must completely match, and four mismatches in the internal regions of the primer sequences were allowed. We compared all of the sequences to the V3ref using GAST (Huse et al. 2008). The remaining sequences were called sequence tags.

### Analysis of the quality-filtered V3 pyrosequencing reads

We performed the species classification by aligning the sequence tags to the V3ref database using BLASTN, and we selected the best alignments as the unique tag aligning results. Unique tags that did not display a single BLAST hit in the V3ref database were also annotated as NA.

To better estimate the diversity of the samples, we sorted the unique tags according to their abundance and performed a preliminary clustering of the data in accordance with a distance of 0.02. The operational taxonomic units (OTUs) were clustered based on a 97 % sequence identity criterion. We performed the OTUs analysis based on the overlapped sequence tags in the CD-HIT program.

### 16S rDNA V3 sequence diversity analysis

Based on the taxonomic composition results with the abundance information, the microbial diversity was evaluated within the samples according to the results of the abundance of the OTUs. A profiling table was constructed by displaying the quantity of the sequence tags of identical classification level. From the profiling table, the abundance differences of the identical species and the species diversity in the different samples were obtained. A beta diversity value closer to 1 indicates a greater species diversity between the different samples.

### Statistical analysis

The results were statistically analyzed using the SPSS 18.0 software package. The Chi-square test was used to analyze the top ten most abundant genera of the three stomachs. The differences were considered to be statistically significant when the *p*-value was less than 0.05.

## Results

### Sequencing and quality control

**Fig. 1:**
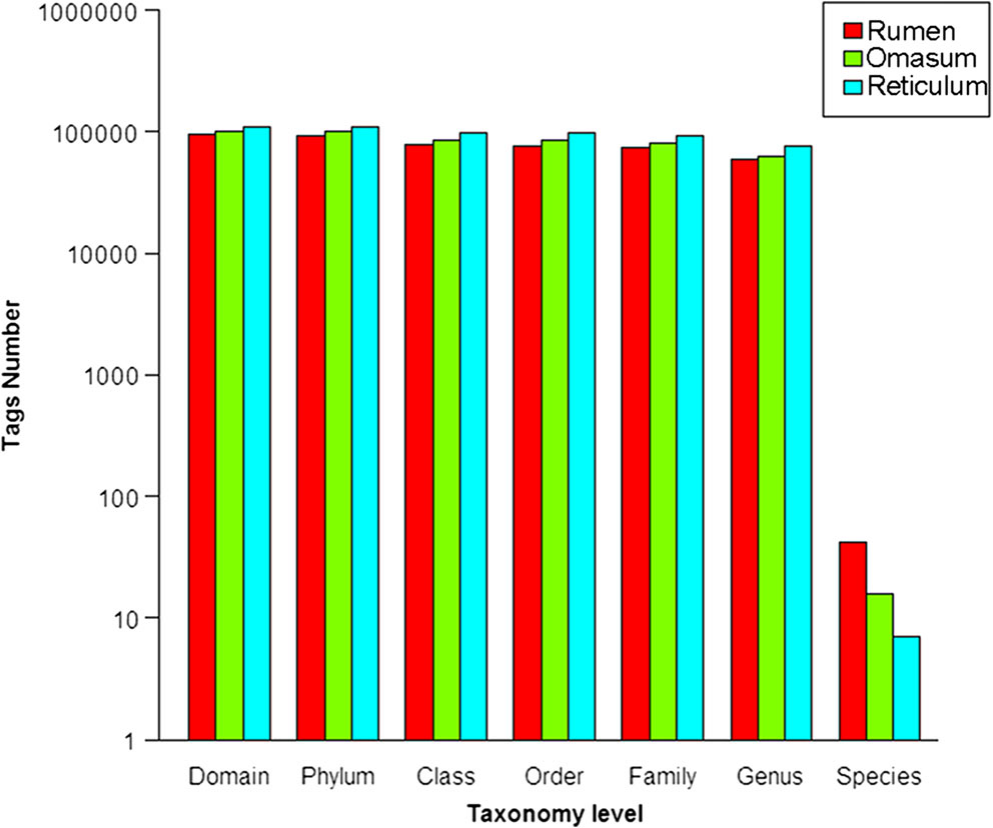
The number of tags at the different taxonomy levels obtained from each sample. Each bar represents the number of tags that were assigned to the taxonomic level for the identified bacteria

Sequencing of the DNA samples purified from the contents of the rumen (Sample 1), the omasum (Sample 2), and the reticulum (Sample 3) generated 1.20, 1.20, and 1.17 gigabases of raw sequence, respectively. Due to a technical difficulty, the DNA from the abomasum was unexpectedly degraded during the sample processing and was not further analyzed in this study. The length of the sequence tags was distributed between 131 and 158 bp, which was determined by the variability of the V3 region of the bacteria, and the average length of the single reads was 152 bp (Supplementary Fig. 1). After preprocessing by removing the low-quality reads and the primer sequences, the total number of sequences obtained in the three samples were 94,682 (14.34 Mb), 101,615 (15.30 Mb), and 110,271 (16.41 Mb), respectively. We were able to classify the majority (more than 98 %) of the sequences below the domain level, and more than 60 % of the tags were assigned to a genus. However, only select sequence tags could be classified at the species level (Fig. 1).

After merging the sequence tags using the mothur software package with redundancy processing, 29,822, 29,540, and 31,827 unique sequence tags were obtained for Samples 1, 2, and 3, respectively (Supplementary Table 1). Then, the abundance of the unique tags was statistically analyzed, and the most unique tags were composed of only one sequence.

### Data indices

The clean sequence tags were clustered into OTUs using a two-stage clustering (TSC) algorithm (Jiang et al. 2012); unique tags were clustered at a distance of 0.02. Sequence tags with 97 % similarity were grouped into OTUs, and 4,283, 4,063, and 4,405 OTUs were obtained from the three samples, respectively. The majority of the OTUs contained only one sequence tag. However, according to the alpha diversity metrics, the omasum was also characterized by a higher npshannon entropy and lower Simpson index. These values indicate that the omasum contained a more diverse microbial community than the other stomach components (Supplementary Table 2). Additionally, the beta diversity was calculated, which indicates the degree of diversity discrepancy in the different samples (Supplementary Table 3).

### Phylum-level taxonomic distribution of the microbiomes in the three stomachs

**Fig. 2:**
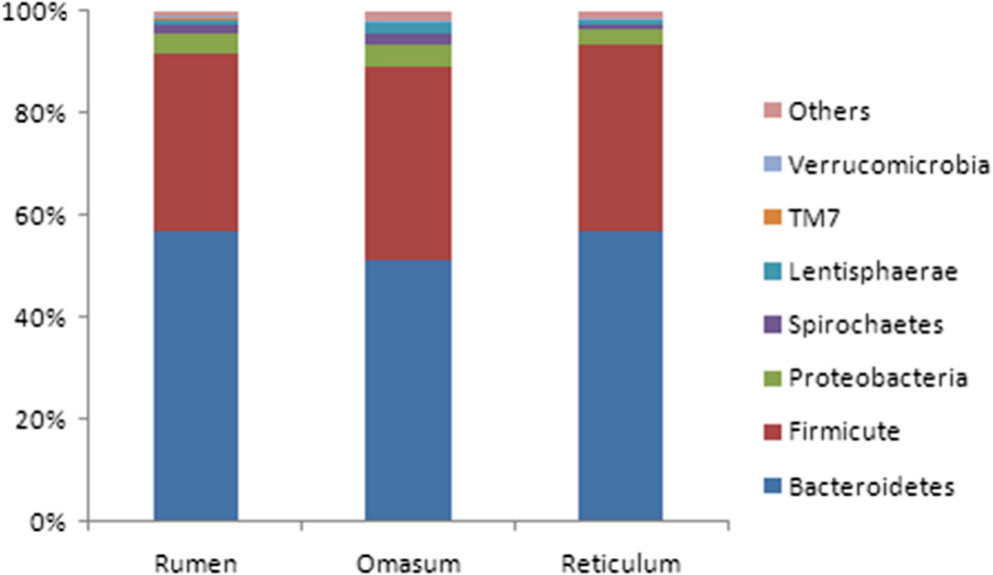
The relative distribution of the seven most abundant phyla in the three stomachs. The percentage of the 16S V3 sequences assigned to a given phylum is represented by the color-coded bars. The seven most abundant phyla representing more than 98 % of the bacteria identified in the three samples are presented separately, whereas the remaining phyla are indicated as “others”

We compared each unique sequence tag to the sequences in the 16S rDNA V3 database (http://vamps.mbl.edu/resources/databases.php) using BLASTN, and the sequence tags that could not be assigned to a phylum were annotated as NA. The V3 sequences were assigned to 17 phyla, and the majority of sequences were assigned to five phyla, Bacteroidetes (56 %), Firmicutes (35 %), Proteobacteria (5.5 %), Spirochaetes (1.6 %), and Lentisphaerae (1.1 %), with various bacterial compositions in the three samples (Table 1 and Fig. 2). The three stomach compartments harbored similar lineages of bacterial phyla, and a total of 13 phyla were assigned in the rumen, 16 in the omasum, and 16 in the reticulum.

**Table 1:** Phylum abundance of bacteria identified in the three samples

Phylum	Rumen	Omasum	Reticulum	Total
Bacteroidetes	57 %	51 %	57 %	55 %
Firmicutes	35 %	38 %	36 %	36 %
Proteobacteria	3.9 %	4.4 %	3.0 %	3.8 %
Spirochaetes	1.6 %	2.0 %	0.9 %	1.5 %
Lentisphaerae	1.2 %	2.2 %	0.9 %	1.4 %

### Subphylum-level distribution of the microbiomes in the three stomach compartments

**Fig. 3:**
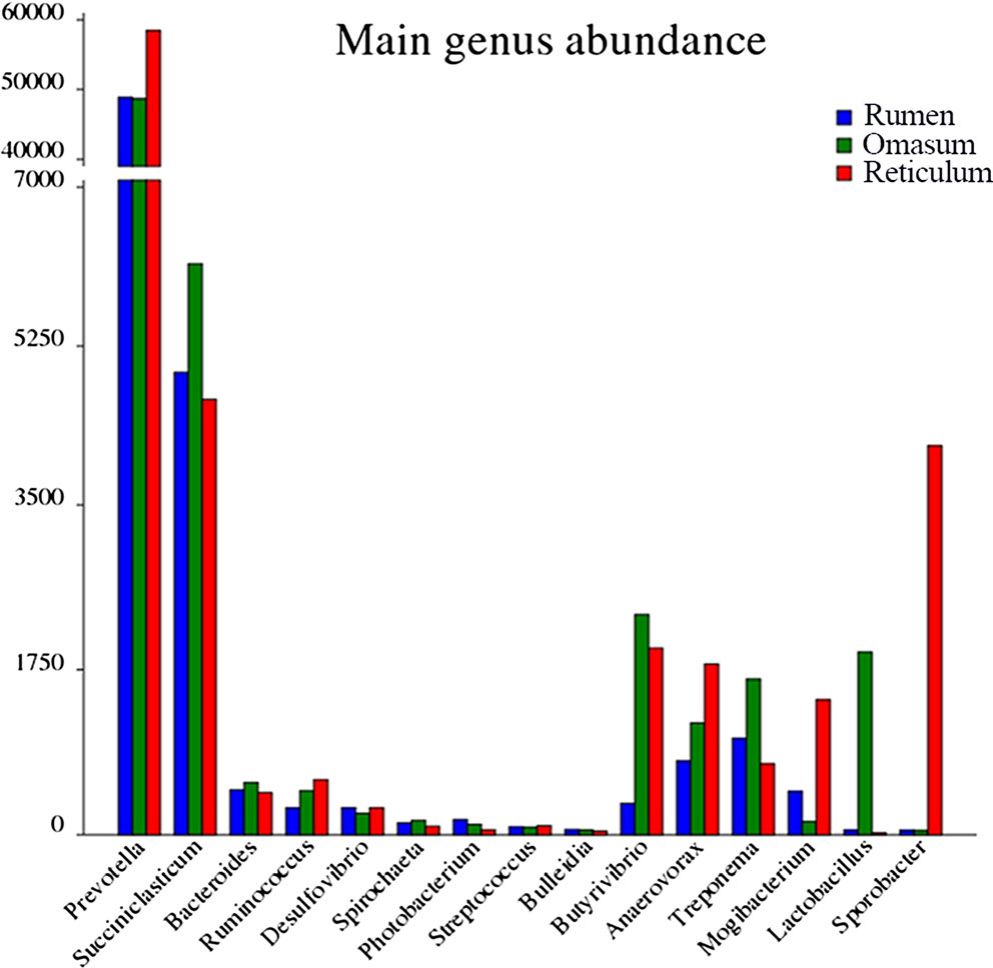
The distribution of the microbiota identified in the three stomachs at the genus level. Each bar represents the number of sequence tags of a bacterial genus from the sample

Bacteria assigned to *Bacteroidetes* were the most predominant in the three samples, and the data obtained from the rumen were similar to previous studies (Fig. 2) (Li et al. 2012a). More than 99 % of the sequence tags of this phylum were assigned to the class Bacteroidia, and the remaining sequence tags were assigned to the class Flavobacteria. Bacteria assigned to Sphingobacteria (36 sequences) were detected only in the omasum. Furthermore, sequences of the Bacteroidetes sequence tags were assigned to five genera. Of these, *Prevotella* was the most predominant genus, which accounted for 51.9 % of all of the bacterial sequence tags. Additionally, 91.2 % of the sequence tags assigned to Bacteroidetes were amplified from *Prevotella* (Fig. 3). However, within the order Bacteroidales, 1,984, 1,416, and 1,736 sequence tags could not be assigned to an existing class, family, or order. Additionally, 1,665, 1,454, and 1,213 sequence tags within the family Prevotellaceae could also not be assigned to an existing genus.

**Fig. 4:**
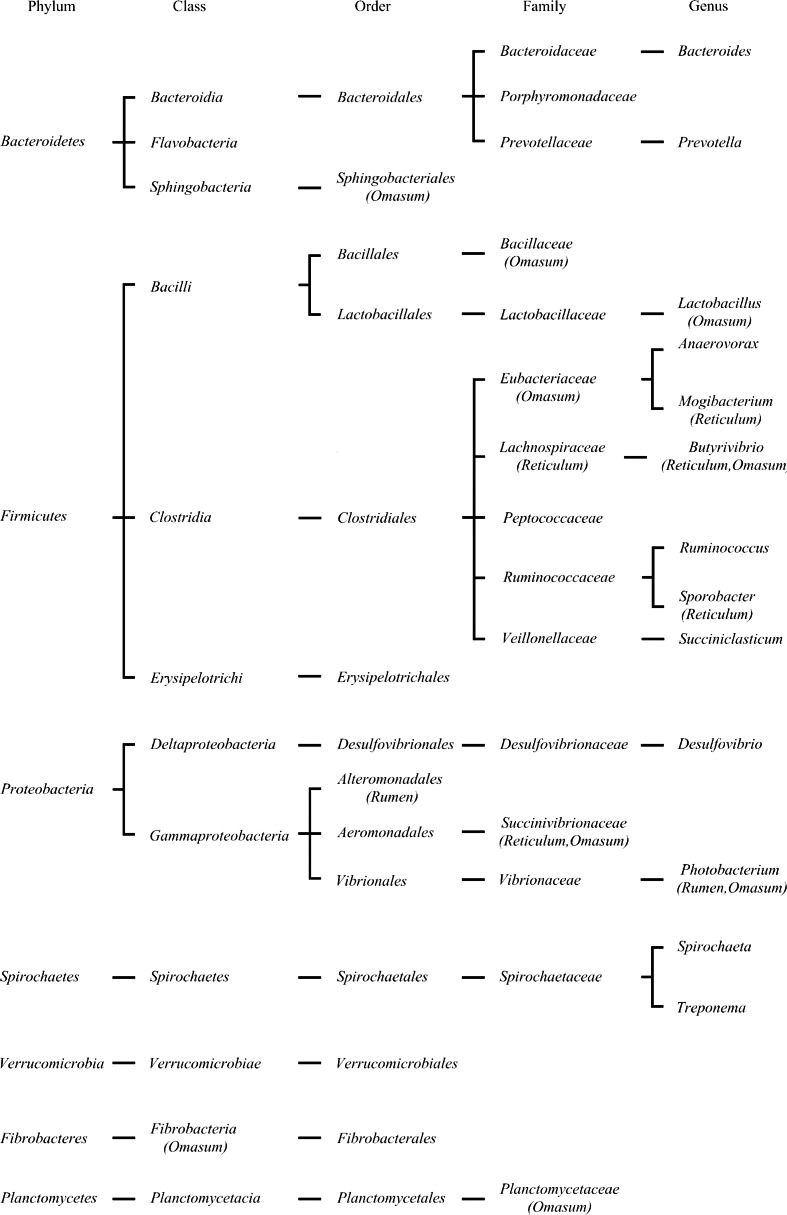
The taxonomy of the microbiota identified from phylum to genus in the three stomachs. The bacteria that were only identified in one or two stomachs are indicated underneath each taxonomy in parentheses

The second most prominently represented sequence tag was from the phylum Firmicutes, and approximately 55.5 %, 63.5 %, and 76.8 % of the sequence tags were assigned to the class Clostridia from the rumen, the omasum, and the reticulum, respectively; the remaining sequence tags were assigned to Bacilli and Erysipelotrichi. All of the sequence tags assigned to Firmicutes were assigned to a total of 48 known genera, and 25 of these genera were within the class Clostridia (Fig. 4 and Supplementary dataset 1). Four families, Ruminococcaceae, Lachnospiraceae, Eubacteriaceae, and Veillonellaceae, were the most abundant families within the class Clostridia (Supplementary Fig. 2). In the family Veillonellaceae, the majority of the sequence tags were assigned to the genus *Succiniclasticum*. Except for *Butyrivibrio*, *Anaerovorax*, *Mogibacterium*, *Ruminococcus* and for *Succiniclasticum*, the other genera were each represented by fewer than 50 sequence tags for each sample within the class Clostridia. Moreover, *Sporobacter* was more abundant in the reticulum (13.5 % of the class Clostridia) than in the other samples (less than 0.01 % of the class Clostridia). The genus *Lactobacillus* within the class Bacilli was the predominant genus (74 % of Bacilli) and was found only in the omasum.

Within the phylum Proteobacteria, all five known classes were found in the three samples. Deltaproteobacteria was the most predominant class within this phylum, which was represented by approximately 59.7 %, 63.5 %, and 70.3 % of all the phylum sequence tags for the three samples. Desulfovibrionaceae was the most predominant family (95.8 %, 97.8 %, and 97.5 % of all of the Deltaproteobacteria sequence tags) (Supplementary dataset 1). Additionally, *Desulfovibrio* was the most predominant genus for the three samples within this class. The second largest class within Proteobacteria was Gammaproteobacteria.

**Fig. 5:**
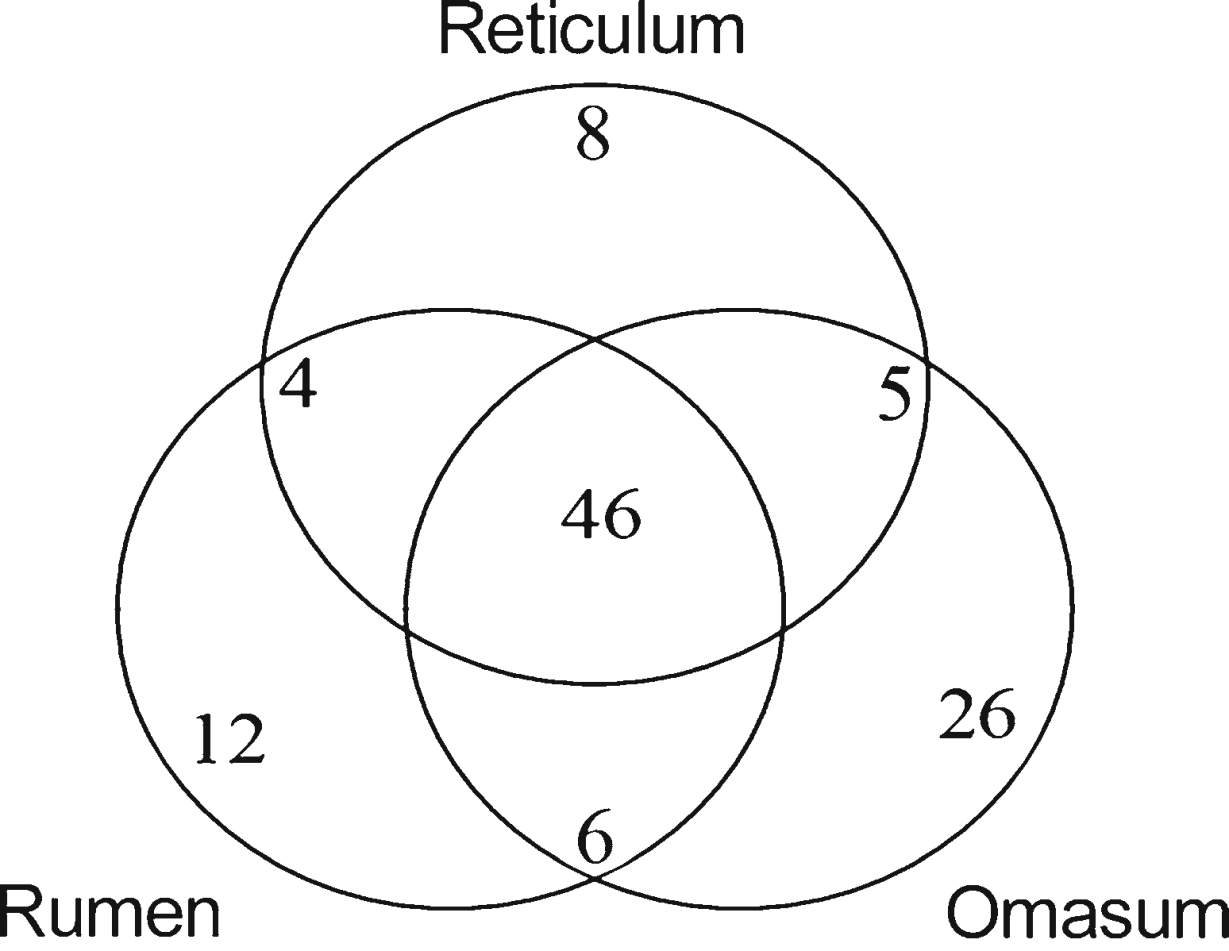
The microbiome shared by the stomachs at the genus level. The number of genera unique to each stomach or shared by two or three stomachs is indicated

Following further analyses of the sequences in the three samples, among the bacteria taxa identified (68, 83, and 63 genera in Samples 1, 2, and 3, respectively), 46 genera were detected in all three samples (Fig. 5 and Supplementary dataset 1). Six taxa were shared between the samples from the rumen and the omasum. Bacteria from the four taxa were shared in samples from the rumen and the reticulum. Five taxa were shared by samples from the omasum and the reticulum. Twelve taxa were found only in the rumen sample, and 26 taxa were found only in the omasum sample. Eight taxa exclusive to the reticulum sample were also detected (Fig. 4 and Supplementary Table 4).

**Fig. 6:**
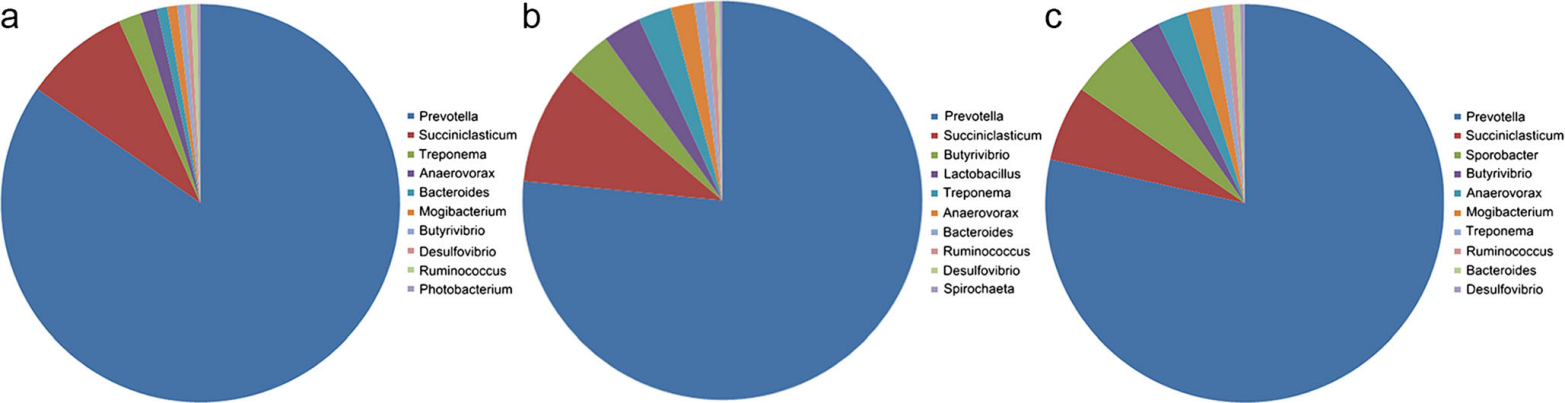
The distribution of top ten most abundant genera in the three stomachs: **a** rumen, **b** omasum, and **c** reticulum

*Prevotella* was the most prevalent genus and *Succiniclasticum* was the second most prevalent. After *Succiniclasticum*, the most abundant taxa were different in the different stomachs (Fig. 6). *Lactobacillus* was more abundant in the omasum than in the other two samples. Additionally, *Sporobacter* was more abundant in the reticulum than in the other two samples. In the omasum and the reticulum, the genus *Butyrivibrio* displayed a higher abundance than in the rumen sample (Fig. 6).

The top ten most abundant genera of the three stomachs were compared, and a significant difference was observed. The *p*-values of all of the top ten most abundant genera were less than 0.05 (*p* < 0.05). However, a difference was not observed in many genera that were not in the top ten most abundant genera, i.e., the genus *Bulleidia*: *p* = 0.430 in the rumen compared with the omasum, *p* = 0.534 in the rumen compared with the reticulum, and *p* = 0.438 in the omasum compared with the reticulum. The differences were also observed at the main class, order, and family level (data not shown).

## Discussion

The stomachs of ruminants are distinct bioreactors characterized by their anatomical structures and complex microbial composition in each compartment. Determining the composition of the microorganisms in the stomach will not only assist in the understanding of ruminant physiology but also facilitate further development of animal husbandry. Studies have concentrated on the identification of microorganisms (bacteria) in the rumen (Edwards et al. 2004; Li et al. 2012b; Stevenson and Weimer 2007; Tajima et al. 1999), but little focus has been placed on the three other stomachs. In the present study, we examined the bacterial composition of the three bovine stomachs by scanning the V3 sequence tag of the 16S ribosomal RNA gene using the Illumina genome analyzer platform. However, as far as we know, this is the first study using high-throughput technology to investigate the microbiota diversity of the reticulum and the omasum. Approximately 38 %, 37 %, and 31 % of the V3 sequence tags obtained from the three samples fell into the category of unclassified genera, showing that the three samples contain many bacteria that have been uncharacterized in previous studies and the microbial diversity of the three samples are much greater than we previously imagined. The sequences from 17 bacterial phyla were identified, and all of the 17 phyla were reported in previous rumen microbiome studies (Kim et al. 2011; Lee et al. 2012; Li et al. 2012a; Tajima et al. 1999), which suggests that these phyla, especially Bacteroidetes and Firmicutes, play a critical role in the rumen. While only 13 phyla were detected in the rumen, 16 phyla were identified in the reticulum and the omasum.

Bacteroidetes was the most abundant phylum in the rumen sample, and the results were similar to that of several other studies (Li et al. 2012a, b; Stevenson and Weimer 2007). The Bacteroidetes populations were greater in the reticulum than in the rumen and the omasum. Bacteroidetes, which is primarily a Gram-negative bacteria (Holdeman et al. 1984), helps to digest starch within the rumen (Stevenson and Weimer 2007). The differences between these studies were likely due to the differences in the PCR primers used (Edwards et al. 2004), the fractions (liquid vs. solid) of the rumen samples that were analyzed, and the feeding environments or food supplies of the sampled animals. It is clear that the food supply plays an important role in the ratio of Firmicutes to Bacteroidetes (Ramirez et al. 2012). While we do not know the implications of the ratio shift, the higher ratio of Firmicutes to Bacteroidetes in stool samples has been associated with increases in weight gain in humans (Ley et al. 2006), and a recent study implied that the frequency of particular microbial phylotypes in the progeny of cattle may be influenced by the sire breed when using different diets (Hernandez-Sanabria et al. 2013). In all three samples, the phylum Bacteroidetes was the most abundant bacterial group. Previous studies reported that the 16S rRNA gene copies of *Prevotella* species far exceeded the others (Avguštin et al. 1997; Stevenson and Weimer 2007; Tajima et al. 2001). A similar result was obtained from this study. Some of the species in *Prevotella*, i.e., *Prevotella ruminicola*, are efficient hemicellulose, cellulose, pectin, long-chain carbohydrate, and protein digesters (Dehority 1969; Nagaraja and Titgemeyer 2007; Owens et al. 1998), which implies their important role in digestion. The results of previous research quantifying *Prevotella* using qPCR have varied both within and between studies, but have consistently found this genus to be highly abundant in the rumen (Stevenson and Weimer 2007; Stiverson et al. 2011). The success of *Prevotella* in the rumen is likely related to the diversity of the species and the functions of this genus within the rumen ecosystem.

In the phylum Firmicutes, there were 48 genera detected in the three samples that were more abundant than the other phyla. The most predominant genus was *Succiniclasticum*, which was the second most dominant genus of all of the 108 genera in the three samples. The genus *Succiniclasticum* is characterized by not being able to ferment carbohydrates, amino acids, or mono-, di-, and tricarboxylic acids other than succinate (which is converted to propionate). Members of this genus are not proteolytic, do not produce a catalase or urease, and do not produce a reduced nitrate. Some species of the genus *Succiniclasticum*, i.e., *Succiniclasticum ruminis*, appear to occur in high numbers (at least 10^8^ cells per gram of ingesta) in the rumen of bovines fed with diets containing grass silage as the main roughage source and in the rumen of bovines at pasture. The level of succinate production in the rumen of bovines fed a grass silage diet is high, which is most likely due to the higher proportion of *Prevotella ruminicola*, a succinate producer, in the rumen than in the other compartments (van Gylswyk 1995). Unsurprisingly, *S. ruminis* and *P. ruminicola* were predominantly found in the rumen.

A significant difference at the genus level was observed among the three samples, especially for the ten most predominant genera in the three stomachs (Fig. 6). The function and physiological environments of the three stomach compartments may be the selective factors operating on the bacterial species. The genus *Treponema*, one of the core members of the rumen bacterial community (Bekele et al. 2011), was identified in all three stomachs and was one of the top ten abundant genera in all three samples, but the abundance of the bacteria of this genus in the three stomachs was significantly different (*p* < 0.05). Although it was the third most abundant genus in the rumen and the fifth most abundant genus in the omasum, the quantity observed in the omasum was much greater than that in the rumen. All of the spirochetes strains isolated from the rumen have been assigned to the genus *Treponema*, which consists of three described species: *Treponema bryantii* (Stanton and Canale-Parola 1979), *Treponema saccharophilum* (Paster and Canale-Parola 1985), and *Treponema zioleckii* (Piknova et al. 2008). It has been reported that bacteria from the *Treponema* strains in the rumen are able to degrade plant polysaccharides from hay or from a concentrated diet (Avguštin et al. 1997; Ziołecki 1979); therefore, it is reasonable to find this group of bacteria in the rumen. The genus *Butyrivibrio* displayed fewer sequences in the rumen than in the other two stomachs. This genus plays a critical role in the decomposition of urea, protein, hemicellulose, and cellulose, and it provides the required ammonia for the growth of the rumen microbial species, especially the bacteria that digest complex carbohydrates (Dehority 1969; Wojciechowicz et al. 1982). Recent research on the rumen microbiome and cattle feed efficiency suggested that the varied functions of the different microbial species may have different impacts on the host rumen fermentation processes (Zhou and Guan 2014). However, little is known regarding the function of these microbes in the reticulum and omasum. Therefore, further work is required to determine the different biological and physiological functions and the reason for the differences in the microbiomes among the three stomachs.

The significant difference in the top ten most abundant genera among the microbial communities in the three stomachs implies that the composition of microbiota within the different stomachs of bovines exhibits considerable heterogeneity. This heterogeneity could come from the different biological and physiological functions of the rumen, the reticulum, and the omasum. The data from this study will facilitate the identification of more bacterial species in ruminant stomachs.

In conclusion, the microbiome composition and diversity in the three stomach compartments of bovines were investigated, and we compared the similarities and differences between the microbiota among the rumen, reticulum, and omasum. This study is among the first to survey the three stomachs of bovine using high-throughput technology. Our findings provide a glimpse of the dazzling microbial diversity of the three stomachs, and the data will undoubtedly facilitate the understanding of host–microbiota mutualism, leading to improvements in animal health and production.

## Electronic supplementary material

Below are the links to the electronic supplementary material.

Supplementary Table 1 The number of sequences and the total length identified from the three samples (DOC 30 kb)

Supplementary Table 2 The alpha diversity of the definite distance OTU (DOC 30 kb)

Supplementary Table 3 The beta diversity of the three stomachs (DOCX 12 kb)

Supplementary Table 4 The distribution of the genera in the three samples (DOCX 13 kb)

Supplementary dataset 1 The distribution of the sequence tags corresponding to the taxonomy. (PDF 71 kb)

Supplementary Fig. 1 The length distribution of the sequence tags. Each bar represents the sum of the tags from each sample (TIFF 109 kb)

High-resolution image (GIF 9 kb)

Supplementary Fig. 2 The most abundant families within the class Clostridia (TIFF 232 kb)

High-resolution image (GIF 25 kb)
