# Supplementary material for: First insights into the microbial diversity in the omasum and reticulum of bovine using Illumina sequencing
Source: J Appl Genet. 2015 Jan 21;56(3):393–401. doi: 10.1007/s13353-014-0258-1 (PMC4543427; doi:10.1007/s13353-014-0258-1)
Supplement: Supplementary file 1 — The number of sequences and the total length identified from the three samples (DOC 30 kb) [file 13353_2014_258_MOESM1_ESM.doc]

| sample |  | Unique tags |  |
| --- | --- | --- | --- |
| ID | Number | Data production (M) | N50 (bp) |
| 1 | 29,882 | 4.50 | 152 |
| 2 | 29,540 | 4.43 | 152 |

Supplementary Table 1 The information of unique tag
