# Supplementary material for: First insights into the microbial diversity in the omasum and reticulum of bovine using Illumina sequencing
Source: J Appl Genet. 2015 Jan 21;56(3):393–401. doi: 10.1007/s13353-014-0258-1 (PMC4543427; doi:10.1007/s13353-014-0258-1)
Supplement: Supplementary file 2 — The alpha diversity of the definite distance OTU (DOC 30 kb) [file 13353_2014_258_MOESM2_ESM.doc]

| *Sample* | *Alpha Diversity* | | |
| --- | --- | --- | --- |
|  | Shannon | npShannon | Simpson |
| 1 | 4.57 | 4.69 | 0.067 |
| 2 | 4.70 | 4.80 | 0.049 |
| 3 | 4.43 | 4.54 | 0.073 |

Supplementary Table 2 Alpha diversity of definite distance OTU
