# Supplementary material for: First insights into the microbial diversity in the omasum and reticulum of bovine using Illumina sequencing
Source: J Appl Genet. 2015 Jan 21;56(3):393–401. doi: 10.1007/s13353-014-0258-1 (PMC4543427; doi:10.1007/s13353-014-0258-1)
Supplement: Supplementary file 3 — The beta diversity of the three stomachs (DOCX 12 kb) [file 13353_2014_258_MOESM3_ESM.docx]

|  | rumen | reticulum | omasum |
| --- | --- | --- | --- |
| rumen | 0 | 0.333 | 0.252 |
| reticulum | 0.333 | 0 | 0.312 |

**Supplementary Table 3 Beta diversity of different stomachs**
