# Supplementary material for: First insights into the microbial diversity in the omasum and reticulum of bovine using Illumina sequencing
Source: J Appl Genet. 2015 Jan 21;56(3):393–401. doi: 10.1007/s13353-014-0258-1 (PMC4543427; doi:10.1007/s13353-014-0258-1)
Supplement: Supplementary file 4 — The distribution of the genera in the three samples (DOCX 13 kb) [file 13353_2014_258_MOESM4_ESM.docx]

**Supplementary Table 2. The distribution of genera in three samples**

|  | Rumen | reticulum | omasum |
| --- | --- | --- | --- |
| *Gemella* | + | + | - |
| *Haemophilus* | + | + | - |
| *Akkermansia* | + | + | - |
| *Anaerotruncus* | + | + | - |
| *Caryophanon* | + | - | + |
| *Coprococcus* | + | - | + |
| *Roseburia* | + | - | + |
| *Subdoligranulum* | + | - | + |
| *Pseudoalteromonas* | + | - | + |
| *Limnobacter* | + | - | + |
| *Oribacterium* | - | + | + |
| *Peptostreptococcus* | - | + | + |
| *Hydrogenophaga* | - | + | + |
| *Bifidobacterium* | - | + | + |
| *Synergistes* | - | + | + |
| *Exiguobacterium* | + | - | - |
| *Jeotgalicoccus* | + | - | - |
| *Aerococcus* | + | - | - |
| *Acidaminococcus* | + | - | - |
| *Erythrobacter* | + | - | - |
| *Pandoraea* | + | - | - |
| *Delftia* | + | - | - |
| *Bdellovibrio* | + | - | - |
| *Lawsonia* | + | - | - |
| *Smithella* | + | - | - |
| *Legionella* | + | - | - |
| *Acinetobacter* | + | - | - |
| *Paenibacillus* | - | + | - |
| *Granulicatella* | - | + | - |
| *Sarcina* | - | + | - |
| *Paracoccus* | - | + | - |
| *Acidovorax* | - | + | - |
| *Kocuria* | - | + | - |
| *Micrococcus* | - | + | - |
| *Sneathia* | - | + | - |
| *Parabacteroides* | - | - | + |
| *Proteiniphilum* | - | - | + |
| *Macrococcus* | - | - | + |
| *Sulfobacillus* | - | - | + |
| *Kurthia* | - | - | + |
| *Peptococcus* | - | - | + |
| *Parvimonas* | - | - | + |
| *Aminobacterium* | - | - | + |
| *Megamonas* | - | - | + |
| *Sphingomonas* | - | - | + |
| *Rhizobium* | - | - | + |
| *Burkholderia* | - | - | + |
| *Diaphorobacter* | - | - | + |
| *Tetrathiobacter* | - | - | + |
| *Bilophila* | - | - | + |
| *Desulfomicrobium* | - | - | + |
| *Actinobacillus* | - | - | + |
| *Mannheimia* | - | - | + |
| *Vibrio* | - | - | + |
| *Escherichia* | - | - | + |
| *Opitutus* | - | - | + |
| *Arthrobacter* | - | - | + |
| *Atopobium* | - | - | + |
| *Phytoplasma* | - | - | + |
| *Anaeroplasma* | - | - | + |
| *Thermus* | - | - | + |

+:sequence tags of the genus were identify

-: sequence tags of the genus were not identify
