# Supplementary material for: First insights into the microbial diversity in the omasum and reticulum of bovine using Illumina sequencing
Source: J Appl Genet. 2015 Jan 21;56(3):393–401. doi: 10.1007/s13353-014-0258-1 (PMC4543427; doi:10.1007/s13353-014-0258-1)
Supplement: Supplementary file 5 — The distribution of the sequence tags corresponding to the taxonomy. (PDF 71 kb) [file 13353_2014_258_MOESM5_ESM.pdf]

									Sample 1	Sample 2	Sample 3
0	domain	Bacteria							785	1161	1027
0	domain	NA							2	7	1
1	phylum		Bacteroidetes						131	113	122
2	class			Bacteroidia					0	0	0
3	order				Bacteroidales				1984	1416	1736
4	family					Bacteroidaceae			263	37	81
5	genus						Bacteroides		481	565	454
6	specy							coprocola	1	0	0
6	specy							fragilis	1	0	0
4	family					Porphyromonadaceae			137	12	147
5	genus						Barnesiella		0	0	0
6	specy							viscericola	36	3	1
5	genus						Parabacteroides		0	1	0
5	genus						Proteiniphilum		0	1	0
4	family					Prevotellaceae			1665	1454	1213
5	genus						Prevotella		49196	48291	59317
2	class			Flavobacteria					0	0	0
3	order				Flavobacteriales				1	0	0
4	family					Flavobacteriaceae			4	3	2
5	genus						Chryseobacterium		1	4	1
2	class			Sphingobacteria					0	0	0
3	order				Sphingobacteriales				0	36	0
1	phylum		Cyanobacteria						18	43	60
2	class			TRUE					0	1	0
1	phylum		Firmicutes						14048	11023	8825
2	class			Bacilli					19	191	1
3	order				Bacillales				37	1	3
4	family					Bacillaceae			5	314	6
5	genus						Bacillus		10	7	5
5	genus						Exiguobacterium		1	0	0
4	family					Caryophanaceae			0	0	0
5	genus						Caryophanon		1	1	0
4	family					Staphylococcaceae			0	0	0
5	genus						Gemella		1	0	1
5	genus						Jeotgalicoccus		1	0	0
5	genus						Staphylococcus		7	10	2
5	genus						Macrococcus		0	1	0
4	family					Alicyclobacillaceae			0	0	0
5	genus						Sulfobacillus		0	1	0
4	family					Planococcaceae			0	0	0
5	genus						Kurthia		0	1	0
4	family					Paenibacillaceae			0	0	0
5	genus						Paenibacillus		0	0	2
3	order				Lactobacillales				15	25	18
4	family					Carnobacteriaceae			5	1	1
5	genus						Carnobacterium		2	7	4
5	genus						Granulicatella		0	0	1
4	family					Streptococcaceae			22	12	16
5	genus						Lactococcus		2	4	1
5	genus						Streptococcus		83	77	95
6	specy							suis	4	3	4
4	family					Aerococcaceae			0	0	0
5	genus						Aerococcus		1	0	0
4	family					Enterococcaceae			0	0	0
5	genus						Enterococcus		5	15	2
5	genus						Tetragenococcus		1	1	2
4	family					Lactobacillaceae			0	0	0
5	genus						Lactobacillus		54	1957	18
6	specy							casei	0	4	0
6	specy							fermentum	0	2	0
6	specy							ruminis	0	4	2
4	family					Leuconostocaceae			0	0	0
5	genus						Leuconostoc		1	2	2

5	genus				Weissella	2	3	1
2	class		Clostridia			415	516	736
3	order			Clostridiales		711	1098	1392
4	family			Clostridiaceae		23	35	19
5	genus				Clostridium	3	8	9
5	genus				Sarcina	0	0	1
4	family			Eubacteriaceae		181	1024	296
5	genus				Anaerovorax	798	1207	1837
5	genus				Eubacterium	1	5	4
5	genus				Mogibacterium	469	145	1458
4	family			Lachnospiraceae		5115	3714	7067
5	genus				Butyrivibrio	340	2372	2010
5	genus				Coprococcus	3	3	0
5	genus				Dorea	5	9	7
5	genus				Roseburia	2	1	0
5	genus				Oribacterium	0	1	1
4	family			Peptococcaceae		242	413	320
5	genus				Peptococcus	0	1	0
4	family			Peptostreptococcaceae		8	13	22
5	genus				Parvimonas	0	1	0
5	genus				Peptostreptococcus	0	1	1
4	family			Ruminococcaceae		4090	6487	5431
5	genus				Anaerotruncus	1	0	1
5	genus				Papillibacter	43	1	1
5	genus				Ruminococcus	291	477	595
5	genus				Sporobacter	49	47	4189
5	genus				Subdoligranulum	1	1	0
4	family			Syntrophomonadaceae		63	71	102
5	genus				Syntrophomonas	4	3	2
5	genus				Aminobacterium	0	1	0
4	family			Veillonellaceae		513	627	615
5	genus				Acidaminococcus	1	0	0
5	genus				Dialister	2	1	1
5	genus				Selenomonas	2	2	6
5	genus				Succiniclasticum	4981	6148	4690
5	genus				Megamonas	0	1	0
2	class		Erysipelotrichi			0	0	0
3	order			Erysipelotrichales		0	0	0
4	family			Erysipelotrichaceae		270	290	193
5	genus				Bulleidia	58	52	41
5	genus				Catenibacterium	2	5	1
5	genus				Turicibacter	21	26	16
1	phylum	Lentisphaerae				1127	2331	1010
1	phylum	Proteobacteria				28	26	17
2	class		Alphaproteobacteria			13	21	17
3	order			Rhodospirillales		0	0	0
4	family			Acetobacteraceae		2	2	3
3	order			Sphingomonadales		0	1	0
4	family			Erythrobacteraceae		0	0	0
5	genus				Erythrobacter	1	0	0
4	family			Sphingomonadaceae		0	0	4
5	genus				Sphingomonas	0	2	0
3	order			Rhizobiales		0	0	3
4	family			Rhizobiaceae		0	0	0
5	genus				Rhizobium	0	2	0
3	order			Rhodobacterales		0	0	0
4	family			Rhodobacteraceae		0	1	0
5	genus				Paracoccus	0	0	1
2	class		Betaproteobacteria			1	0	0
3	order			Burkholderiales		2	2	0
4	family			Burkholderiaceae		2	1	0
5	genus				Limnobacter	2	1	0
5	genus				Pandoraea	1	0	0
5	genus				Burkholderia	0	1	0

4	family	Comamonadaceae	37	72	52
5	genus	Comamonas	3	1	5
5	genus	Delftia	1	0	0
5	genus	Diaphorobacter	0	1	0
5	genus	Hydrogenophaga	0	1	2
5	genus	Acidovorax	0	0	1
4	family	Oxalobacteraceae	1	0	4
4	family	Unassigned	0	0	0
5	genus	Aquabacterium	1	2	1
4	family	Alcaligenaceae	0	0	1
5	genus	Tetrathiobacter	0	1	0
3	order	Neisseriales	0	0	0
4	family	Neisseriaceae	3	10	2
2	class	Deltaproteobacteria	11	15	6
3	order	Desulfuromonadales	35	22	25
4	family	Geobacteraceae	0	1	0
3	order	Bdellovibrionales	0	0	0
4	family	Bdellovibrionaceae	0	0	0
5	genus	Bdellovibrio	29	0	0
3	order	Desulfobacterales	0	0	0
4	family	Desulfobulbaceae	0	2	2
5	genus	Desulfobulbus	15	11	19
4	family	Desulfobacteraceae	0	0	1
3	order	Desulfovibrionales	0	0	0
4	family	Desulfovibrionaceae	1822	2559	1994
5	genus	Desulfovibrio	293	233	291
5	genus	Lawsonia	1	0	0
5	genus	Bilophila	0	1	0
4	family	Desulfomicrobiaceae	0	0	0
5	genus	Desulfomicrobium	0	1	0
3	order	Syntrophobacterales	0	0	0
4	family	Syntrophaceae	0	0	1
5	genus	Smithella	1	0	0
3	order	Myxococcales	0	6	2
4	family	Polyangiaceae	0	1	2
2	class	Gammaproteobacteria	1095	1264	694
3	order	Alteromonadales	91	1	11
4	family	Pseudoalteromonadaceae	0	0	0
5	genus	Pseudoalteromonas	5	4	0
4	family	Shewanellaceae	0	0	0
5	genus	Shewanella	3	2	1
3	order	Aeromonadales	0	0	0
4	family	Succinivibrionaceae	1	80	72
3	order	Legionellales	0	0	0
4	family	Legionellaceae	0	0	0
5	genus	Legionella	1	0	0
3	order	Pasteurellales	0	0	0
4	family	Pasteurellaceae	7	2	6
5	genus	Haemophilus	1	0	1
5	genus	Actinobacillus	0	1	0
5	genus	Mannheimia	0	1	0
3	order	Pseudomonadales	0	0	0
4	family	Moraxellaceae	0	0	0
5	genus	Acinetobacter	2	0	0
3	order	Vibrionales	0	0	0
4	family	Vibrionaceae	6	3	1
5	genus	Photobacterium	165	113	54
5	genus	Vibrio	0	1	0
3	order	Xanthomonadales	0	0	0
4	family	Xanthomonadaceae	1	1	0
3	order	Enterobacteriales	0	0	0
4	family	Enterobacteriaceae	0	0	0
5	genus	Escherichia	0	1	0
2	class	Epsilonproteobacteria	0	0	0

3 order			Campylobacterales			0	0	0
4 family				Campylobacteraceae		0	0	0
5 genus					Campylobacter	12	15	34
1 phylum	Spirochaetes					25	15	28
2 class		Spirochaetes				0	0	0
3 order			Spirochaetales			0	0	0
4 family				Spirochaetaceae		322	245	53
5 genus					Borrelia	2	5	2
5 genus					Spirochaeta	130	154	91
5 genus					Treponema	1042	1678	767
1 phylum	TM7					173	326	464
1 phylum	Verrucomicrobia					5	19	5
2 class		Verrucomicrobiae				0	0	0
3 order			Verrucomicrobiales			203	157	102
4 family				Akkermansiaceae		0	0	0
5 genus					Akkermansia	1	0	1
4 family				Verrucomicrobiaceae		0	0	1
2 class		Opitutae				0	0	0
3 order			Opitutales			0	0	0
4 family				Opitutaceae		0	0	0
5 genus					Opitutus	0	2	0
1 phylum	Euryarchaeota					0	0	0
1 phylum	Actinobacteria					0	0	0
2 class		Actinobacteria				4	12	7
3 order			Actinomycetales			13	23	17
4 family				Microbacteriaceae		1	0	1
4 family				Micrococcaceae		5	28	4
5 genus					Arthrobacter	0	1	0
5 genus					Kocuria	0	0	1
5 genus					Micrococcus	0	0	2
4 family				Nocardiopsaceae		1	0	0
4 family				Corynebacteriaceae		0	0	0
5 genus					Corynebacterium	4	11	10
4 family				Dietziaceae		0	0	0
5 genus					Dietzia	1	2	3
4 family				Streptomycetaceae		0	0	0
5 genus					Streptomyces	1	2	1
4 family				Nocardiaceae		0	1	0
4 family				Propionibacteriaceae		0	1	0
3 order			Coriobacteriales			0	0	0
4 family				Coriobacteriaceae		3	6	12
5 genus					Eggerthella	9	10	11
5 genus					Atopobium	0	1	0
3 order			Acidimicrobiales			0	1	0
3 order			Bifidobacteriales			0	0	0
4 family				Bifidobacteriaceae		0	1	0
5 genus					Bifidobacterium	0	4	1
1 phylum	Fibrobacteres					0	0	0
2 class		Fibrobacteria				0	0	0
3 order			Fibrobacterales			87	203	36
4 family				Fibrobacteraceae		0	0	0
5 genus					Fibrobacter	1	5	1
1 phylum	Fusobacteria					0	0	0
2 class		Fusobacteria				0	0	0
3 order			Fusobacteriales			0	0	0
4 family				Fusobacteriaceae		0	0	0
5 genus					Fusobacterium	5	4	13
4 family				Leptotrichiaceae		0	0	0
5 genus					Sneathia	0	0	1
1 phylum	Planctomycetes					0	0	0
2 class		Planctomycetacia				0	0	0
3 order			Planctomycetales			0	0	0
4 family				Planctomycetaceae		77	242	38
5 genus					Gemmata	1	1	1

1	phylum	Tenericutes	0	0	0
2	class	Mollicutes	1	3	3
3	order	Acholeplasmatales	0	0	0
4	family	Acholeplasmataceae	0	0	0
5	genus	Phytoplasma	0	2	0
3	order	Anaeroplasmatales	0	0	0
4	family	Anaeroplasmataceae	0	0	0
5	genus	Anaeroplasma	0	2	0
1	phylum	Acidobacteria	0	1	0
2	class	Acidobacteria	0	0	0
3	order	Acidobacteriales	0	0	1
4	family	Acidobacteriaceae	0	0	0
5	genus	Gp3	0	0	1
1	phylum	Deferribacteres	0	0	0
2	class	Deferribacteres	0	0	0
3	order	Deferribacterales	0	0	0
4	family	Unassigned	0	0	0
5	genus	Synergistes	0	3	7
1	phylum	Deinococcus-Thermus	0	0	0
2	class	Deinococci	0	0	0
3	order	Thermales	0	0	0
4	family	Thermaceae	0	0	0
5	genus	Thermus	0	2	0
1	phylum	Chloroflexi	0	0	2
