# Supplementary figures and images for: First insights into the microbial diversity in the omasum and reticulum of bovine using Illumina sequencing
Source: J Appl Genet. 2015 Jan 21;56(3):393–401. doi: 10.1007/s13353-014-0258-1 (PMC4543427; doi:10.1007/s13353-014-0258-1)

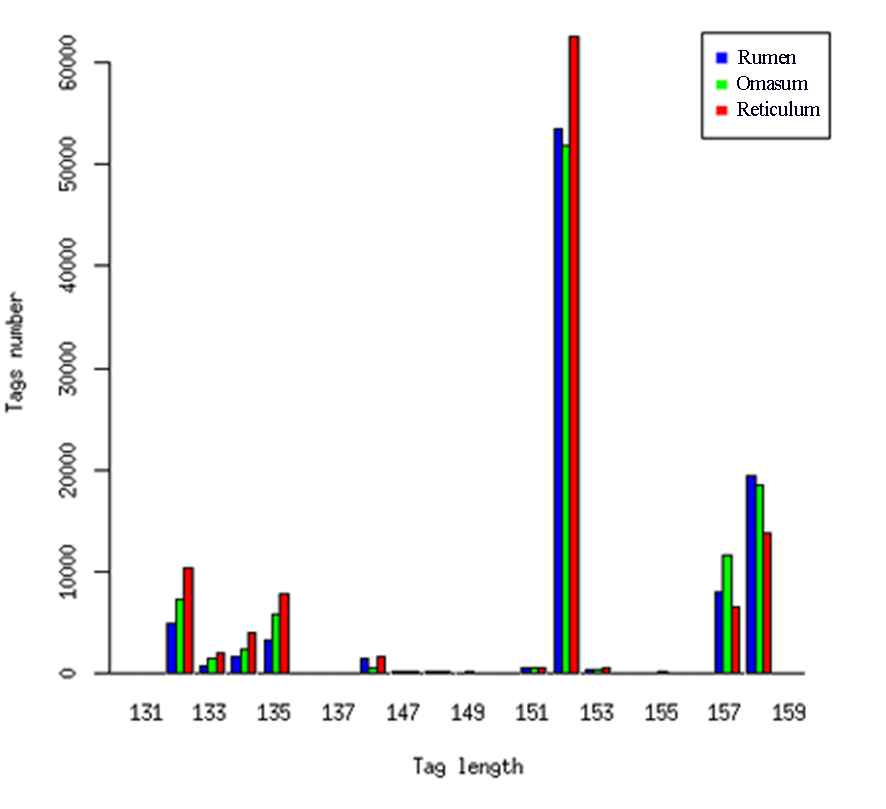

Supplement: Supplementary file 6 — The length distribution of the sequence tags. Each bar represents the sum of the tags from each sample (TIFF 109 kb) [file 13353_2014_258_MOESM6_ESM.tif]

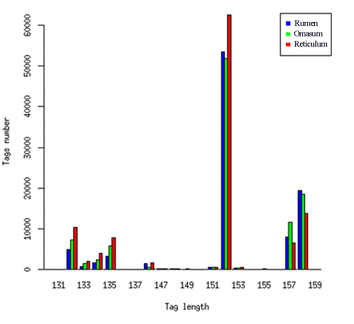

Supplement: Supplementary file 7 — (GIF 9 kb) [file 13353_2014_258_Fig7_ESM.gif]

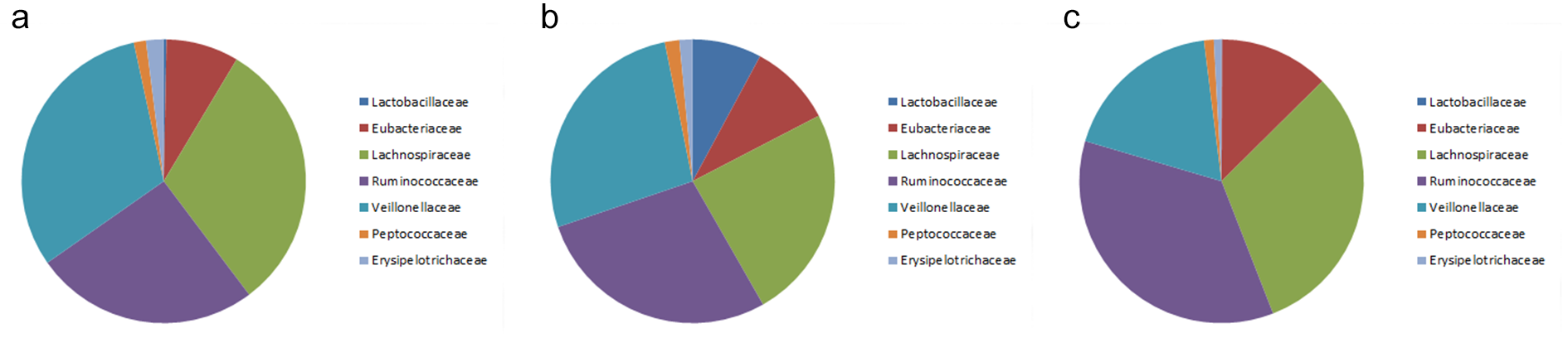

Supplement: Supplementary file 8 — The most abundant families within the class Clostridia (TIFF 232 kb) [file 13353_2014_258_MOESM7_ESM.tif]

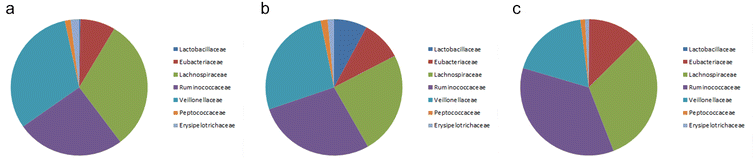

Supplement: Supplementary file 9 — (GIF 25 kb) [file 13353_2014_258_Fig8_ESM.gif]
